# The utility of neutrophil to lymphocyte ratio and fluid sequestration as an early predictor of severe acute pancreatitis

**DOI:** 10.1038/s41598-017-10516-6

**Published:** 2017-09-06

**Authors:** Chaoqun Han, Jun Zeng, Rong Lin, Jun Liu, Wei Qian, Zhen Ding, Xiaohua Hou

**Affiliations:** 1 Department of Gastroenterology, Union Hospital, Tongji Medical College, Huazhong University of Science and Technology, Wuhan, 430022 China; 2 Department of Gastroenterology, the Central Hospital of Wuhan, Tongji Medical College, Huazhong University of Science and Technology, Wuhan, 430014 China

## Abstract

It is important to identify the patients with high-risk progression to develop severe acute pancreatitis (SAP). The study was to assess whether neutrophil to lymphocyte ratio (NLR) and fluid sequestration (FS) could represent useful markers for predicting the severity. A total of 1639 patients who underwent clinical diagnosis of AP was performed. Various serologic and clinical parameters on admission were investigated. Chronologic change in NLR and FS were analyzed, and theirs utility for predicting severity of AP was evaluated by receiver operator characteristic (ROC) curve analysis. Correlation analysis was assessed by Spearman’s rank test. NLR and FS levels were both increased significantly in SAP and positively correlated with Ranson score and hospital stays. The ROC curve analyses showed the optimal cut-off values of NLR for admission with day0, day1, day2 were 9.64, 6.66 and 6.50, giving sensitivity of 77–82%. The optimal cut-off values of FS for admission with day1, day2, day3 were 1375 ml, 2345 ml and 3424 ml, giving sensitivity of 62–75%. Moreover, measurement of NLR and FS together exhibited a similar area under curve (AUC) and sensitivity for SAP prediction compared with the those of Ranson score. Increase of NLR and FS are correlated with severity and can be suggested as a predictive factor in an early stage of AP.

## Introduction

Despite advances of diagnosis and treatment, acute pancreatitis (AP) was still the largest contributor to aggregate costs and the fifth leading cause of in-hospital deaths^1^. The major cause is the infected necrosis, which is associated with a poor prognosis: mortality rate is approximately 15% with severe acute pancreatitis (SAP) and up to 30–50% in those with infectious complications^2–4^. This dismal outcome is partly due to the lack of an effective method for timely predicting the severity of AP at the time of diagnosis.

A series of scoring systems are currently accepted to stratify the severity of AP and the initiation of aggressive early treatment. the Ranson criteria, the Acute Physiology and Chronic Health Evaluation (APACHEII) system and the Bedside Index for Severity in Acute Pancreatitis (BISAP) score are most widely used in clinical practice. However, there is no universally adopted criteria, due to their more parameters, low sensitivity and the complex for quick evaluation^5^. Therefore, novel and simple predictors to complement scoring systems are urgently needed to improve clinical outcomes.

Although current guidelines recommend early fluid therapy in order to prevent tissue hypoperfusion and avert hypoxia, there is no consensus on the optimal amount and rate of fluid resuscitation^6–8^; the evidence on which it is based remains paltry and of poor quality. The need for a great amount of fluid during the initial 24 h was associated with adverse outcome; and caution should be attention with regard to volume therapy in patients with AP^7, 9, 10^. Therefore, it’s still unclear which patients should receive aggressive or nonaggressive fluid administration. Moreover, clinical studies have mainly focused on the quantity of fluid resuscitation over different time points but very few have evaluated the role of fluid sequestration (FS). The identification of role of FS could help modulate fluid resuscitation for each individual patient, with aggressive resuscitation reserved for those with predicted increased fluid requirements or signals of fluid redundance^11^. The balance for FS would be a early alarm for predicting AP outcome.

Additionally, the neutrophil to lymphocyte ratio (NLR) is an easy accessibility of laboratory test that is already presented in predicting poor outcomes in a variety of severe diseases (i.e. bacteremia and gastroentinal neoplasms)^12–14^. Increase of the NLR is also an independent negative prognostic indicator for SAP but it remains a great controversy^15–17^. Yet till now, no previous data have examined whether the combination of NLR and FS can predict the severity of AP upon admission. Therefore, the present study was to investigate the ability of these two useful and simple indexs for assessing occurrence of SAP.

## Results

### Demographic, clinical characteristics, and severity parameters

A total of 328 patients with AP were admitted who met all inclusion criteria of our study. These cases consisted of 215 men and 113 women with a mean age of 47.5 years (range 18–79 years) 0.170 patients had MAP and 158 patients had SAP. Most patients predominately presented with a disease of abdominal pain (59.8%) and the risk factors of AP (included etiologies) were presumed to be alcohol in 114 patients, smoking in 86 patients, gallstone in 56 patients, prior acute pancreatitis in 40 patients and others in 32 patients. Regarding a variety of laboratory indexes, the white blood cell counts (WBC), neutrophil count, lactate dehydrogenase (LDH) and CRP of the SAP group were significantly increased compared to the MAP group on admission (P < 0.01). The SAP cohort had significant high Ranson score and impaired renal function as indicated by higher serum urea nitrogen levels, and lower albumin and calcium levels (P < 0.05). The mean number of hospital days for the SAP patients were also greater than those of the MAP patients (P < 0.01). Hemoglobin and triglyceride were higher in the SAP group but not found to be statistically significant (P > 0.05). There was also no difference between patient age, gender and other serum markers including liver function tests, lymphocyte count or amylase levels (Table 1).

**Table 1:** Comparison of the demographic features and laboratory values according to disease severity on admission between the two group patients.

Variable	All	MAP	SAP	P value
Entire series	328	170	158	
**Demographic characteristics**				
Gender; male (%)	215 (65.5%)	108 (63.5%)	107 (67.7%)	0.486
Age (y)	47.47 ± 13.49	46.54 ± 13.63	48.48 ± 13.30	0.193
**Risk factors n (%)**				
Alcohol	114 (34. 8%)	52 (30.6%)	62 (39.2%)	
Smoking	86 (26.2%)	56 (32.9%)	59 (37.3%)	0.420
Gallstones	56 (17.1%)	27 (15.9%)	29 (18.4%)	0.561
Prior acute pancreatitis	40 (12.2%)	23 (13.5%)	17 (10.8%)	0.501
Others	32 (9.7%)	20 (11.8%)	12 (7.6%)	
**Clinical and laboratory data**				
Abdominal ain	196 (59.8%)	101 (59.4%)	95 (60.1%)	0.451
Concomitant symptoms	132 (40.2%)	69 (40.6%)	63 (39.9%)	0.447
Temperature, °C	36.80 ± 0.60	36.76 ± 0.58	36.85 ± 0.62	0.627
Amylase, U/l	694.67 ± 695.62	578.36 ± 613.94	814.85 ± 754.36	0.107
WBC (G/L)	12.77 ± 5.50	10.89 ± 4.11	14.79 ± 6.06	0.000*
RBC (G/L)	4.59 ± 0.72	4.75 ± 0.76	4.45 ± 0.64	0.158
Hemogobin	141.05 ± 24.50	147.02 ± ± 25.23	135.50 ± 22.47	0.091
PLT	173.81 ± 60.66	177.14 ± 63.26	170.23 ± 57.71	0.346
Neutrophil	11.18 ± 5.08	9.27 ± 3.90	13.22 ± 5.41	0.000*
Lymphocyte	1.02 ± 0.79	1.09 ± 0.64	0.94 ± 0.92	0.570
ALT (IU)	88.97 ± 151.04	82.65 ± 146.49	95.78 ± 155.98	0.601
AST (IU)	76.76 ± 122.91	64.44 ± 112.25	89.94 ± 132.47	0.212
LDH (IU)	338.08 ± 202.76	216.63 ± 47.18	468.75 ± 223.78	0.0001*
RP (mg/L)	152.04 ± 118.45	93.29 ± 73.74	215.25 ± 124.94	0.0007*
Albumin (g/L)	35.71 ± 6.94	37.38 ± 6.22	33.96 ± 7.24	0.021*
Glucose (mmol/L)	9.06 ± 4.72	8.11 ± 4.76	9.94 ± 4.52	0.389
Hematocrit, %	41.31 ± 7.06	42.61 ± 6.51	40.08 ± 7.36	0.293
Calcium (mmol/L)	2.00 ± 0.32	2.13 ± 0.27	1.86±0.32	0.000*
Serum urea nitrogen	5.30 ± 2.59	4.54 ± 1.72	6.12 ± 3.09	0.000*
Triglyceride (mmol/L)	7.48 ± 11.35	6.64 ± 11.02	8.33 ± 11.66	0.186
hospial stay (days)	17.86 ± 13.40	12.92 ± 6.96	23.18 ± 16.33	0.000*
Ranson score	3.39 ± 1.63	2.54 ± 1.37	4.28 ± 1.39	0.000

MAP, mild acute pancreatitis; SAP, severe acute pancreatitis; WBC, white blood cells; RBC, red blood cells; PLT, platelets; ALT, alanine aminotransferase; AST, aspartate transaminase; LDH, lactate dehydrogenase; CRP, C-reactive protein. Concomitant symptoms included abdominal distention, nausea, vomit, diarrhea and jaundice, etc. Others included idiopathic pancreatitis, post-endoscopic retrograde cholangiopancreatography, hereditary, etc. *Statistically significant difference between MAP and SAP groups.

### Neutrophil to lymphocyte ratio (NLR)

**Figure 1:**
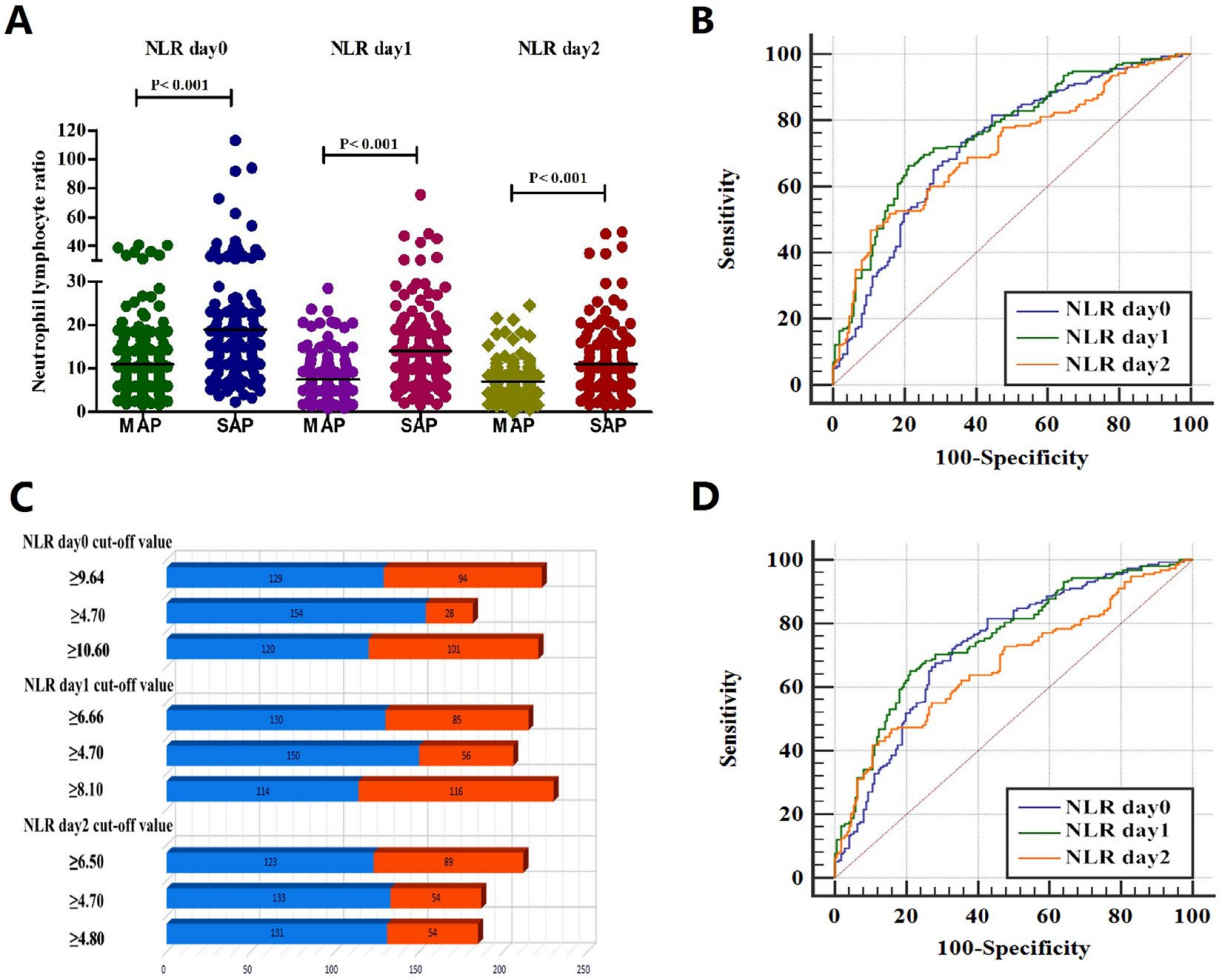
The role of neutrophil to lymphocyte ratio (NLR) in predicting severe of acute pancreatitis. (**A**) The quantitative values of NLR for cohort. Black horizontal lines are means, and error bars are SDs; (**B**). Receiver operator characteristic (ROC) curve of NLR after admission within day0, day1 and day2 for predicting SAP; (**C**). The number of accurate judgment of SAP in patients whose NLR were greater than or equal to cut-off values; (**D**). ROC curve of NLR for predicting SAP included the 11 patients who have been used carbapenem antibiotics (meropenem and imipenem) after admission.

**Figure 2:**
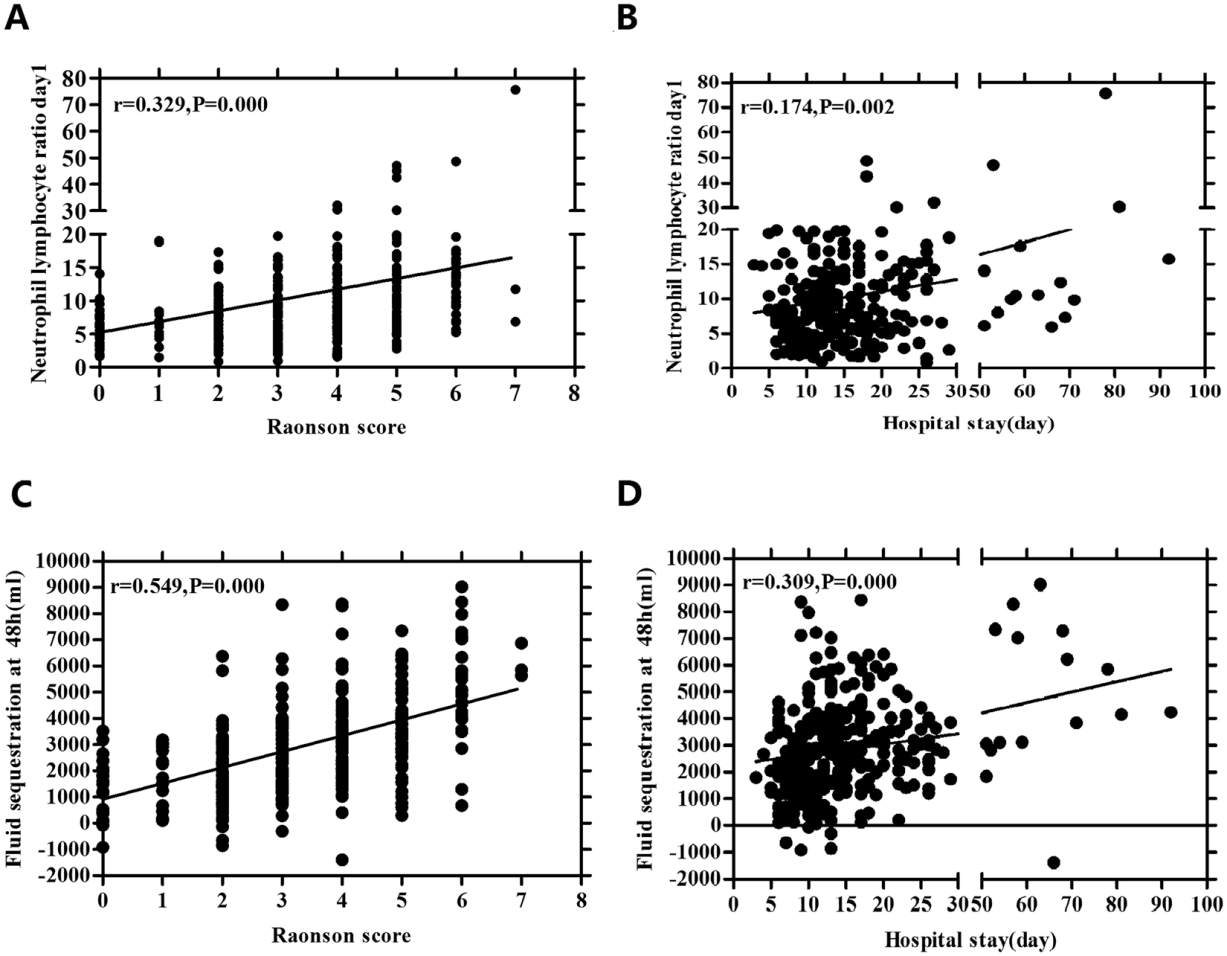
Correlations of neutrophil to lymphocyte ratio (NLR) and fluid sequestration (FS) levels on admission with severity indexes in SAP. (**A**) Correlation NLR with Ranson score; (**B**). Correlation NLR with hosptical stays; (**C**). Correlation FS with Ranson score; (**D**). Correlation FS with hosptical stays.

The mean values of NLR levels in patients with SAP on admission within day0, day1, and day2 were 18.92 ± 15.50, 14.09 ± 9.92, 11.04 ± 8.06, respectively; which were significantly higher than those in the MAP groups (Fig. 1A, P < 0.001). ROC curve analyses were used to evaluate the values for NLR to predict severity in AP patients (Fig. 1B). The AUC values of NLR for day0, day1, and day2 were 0.723 (CI = 0.672–0.771), 0.762 (CI = 0.712–0.807), and 0.711 (CI = 0.659–0.760), respectively (Table 2). ROC curves were also performed to obtain the optimal cut-off levels for each time of this population; and comparison of AUC revealed that the optimal cut-off values of NLR were 9.64, 6.66, 6.50 for day0, day1, and day2, respectively; giving sensitivity of 77–82%, specificity of 50–55%, positive predictive value (PPV) of 60–63% and negative predictive value (NPV) of 71–76% (Fig. 1C; Table 2). When the utility of the NLR on day1 was compared with that of other times by optimal cut-off value, the NLR on admission within 48 h had the highest AUC and the highest sensitivity (Table 2). NLR was also significantly positively correlated with the Ranson score and hospital stays (r = 0.329, P = 0.000; r = 0.174, P = 0.002, respectively; Fig. 2A and B).

**Table 2:** The predictive values of NLR/FS and the Ranson scoring system for AP severity.

Parameter	optimal cut-of value	Sens (%)	Spec (%)	PPV (%)	NPV (%)	AUC (95%CI)	P
NLR-24 h (day0)	9.64	81.65	55.29	62.93	76.43	0.723 (0.672–0.771)	<0.0001
	4.70	97.47	16.47	52.03	87.51		
	10.60	75.95	59.41	63.49	72.66		
NLR-48 h (day1)	6.66	82.28	50.00	60.46	75.22	0.762 (0.712–0.807)	<0.0001
	4.70	94.94	32.94	56.82	87.51		
	8.10	72.15	68.24	67.86	72.50		
NLR-72 h (day2)	6.50	77.85	52.35	60.29	71.78	0.711 (0.659–0.760)	<0.0001
	4.70	84.18	31.76	53.41	68.36		
	4.80	82.91	31.76	53.03	66.66		
FS-24 h (day1)	1375 ml	74.05	51.18	58.50	67.97	0.660 (0.606–0.711)	<0.0001
FS-48 h (day2)	2345 ml	75.32	51.76	59.20	69.29	0.717 (0.665–0765)	<0.0001
FS-72 h (day3)	3424 ml	62.03	57.06	57.31	61.79	0.675 (0.621–0.725)	<0.0001
Ranson score	3	79.75	76.47	75.90	80.25	0.824 (0.778–0.864)	<0.0001

NLR, neutrophil lymphocyte ratio; FS, fluid sequestration; Sens, sensitivity; Spec, specificity; PPV, positive predictive value; NPV, negative predictive value; AUC, area under the curve. CI, confidential interval. An optimal NLR = 4.7 was proposed by Basem Azab *et al*. and other optimal cut-offs NLR = 10.6 (day 0), 8.1 (day 1), and 4.8 (day 2) were suggested by Aravind Suppiah *et al*. Significance level P was compared with Area = 0.5.

Additionally, to verify the influence of carbapenem antibiotics on NLR, we included 11 patients who have been used meropenem and imipenem on admission. The AUC values of NLR for day0, day1, and day2 turned to 0.731 (95% CI = 0.680–0.779), 0.754 (95% CI = 0.704–0.800), and 0.673 (95%CI = 0.620–0.724), respectively (Fig. 1D). These results indicated that NLR was a useful marker for predicting SAP. In particular, the utility of the NLR on admission within 48 h (day1) may be the most effective time point to differentiate patients.

### Fluid sequestration (FS)

**Figure 3:**
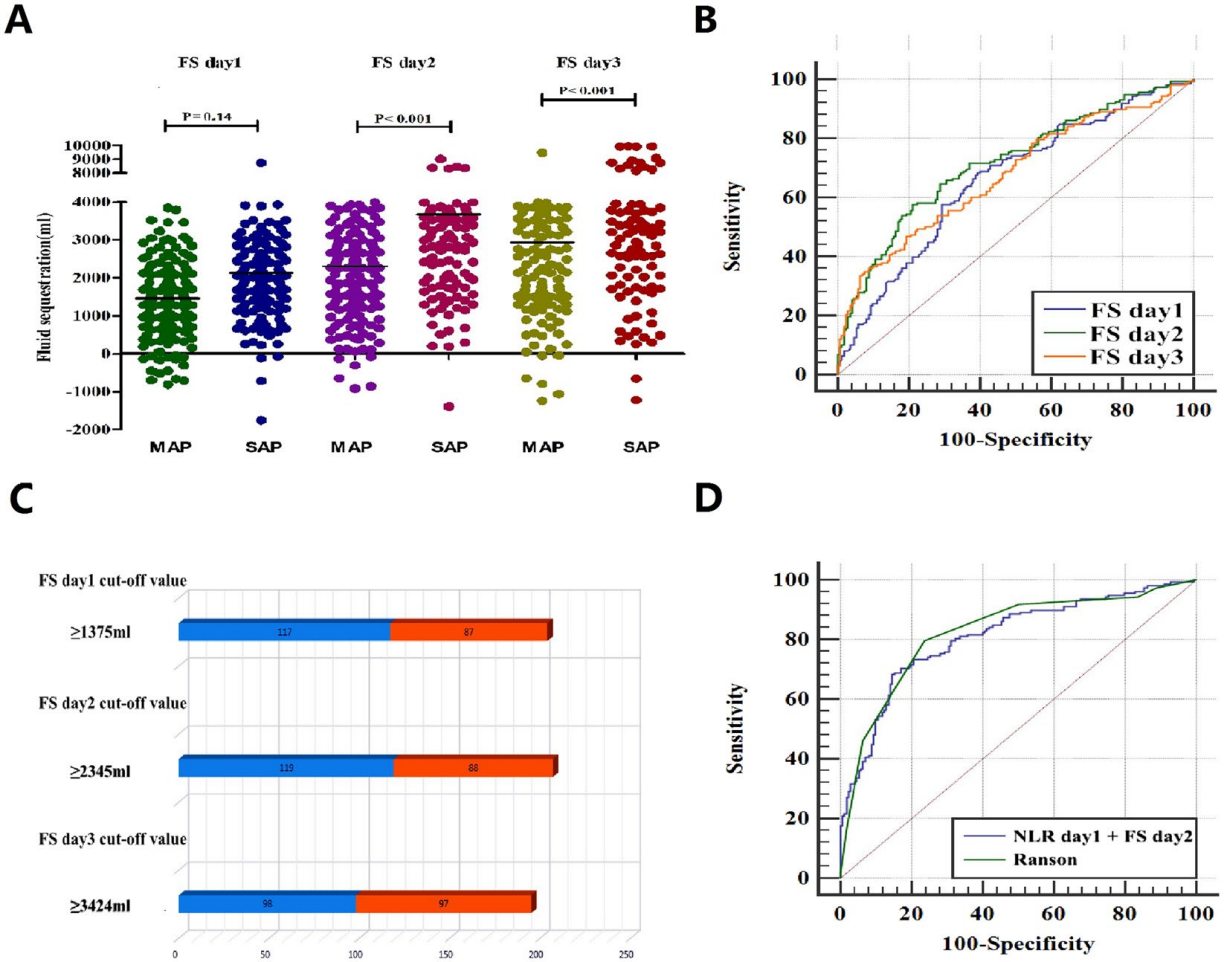
The role of fluid sequestration (FS) in predicting severe of acute pancreatitis. (**A**) The quantitative values of FS for cohort. Black horizontal lines are means, and error bars are SDs; (**B**). Receiver operator characteristic (ROC) curve of FS for day1, day2 and day3 for predicting SAP; (**C**). The number of accurate judgment of SAP in patients whose FS were greater than or equal to cut-off values; (**D**). Association of neutrophil lymphocyte ratio (NLR) and FS with Ranson scoring system by ROC curve analysis.

Next, as a parameter for the acute FS of the host, change of FS and use of FS for the prediction of SAP were investigated. As shown in Fig. 3A, the levels of FS were significantly higher in the SAP groups than those in the MAP groups on admission with day2 and day3 (3677.75 ± 1924.91 vs 2303. 88 ± 1440.13 ml; 4329.88 ± 2415.51 vs 2935.44 ± 1824.25 ml; P < 0.001). The FS for day1 of SAP group had a nonsignificant difference compared to MAP patients (2126 ± 1332.29 vs 1458.85 ± 1043.23 ml, P = 0.140). The AUC of the FS values were 0.660 (CI = 0.606–0.711), 0.717 (CI = 0.665–0.765), 0.675 (CI = 0.621–0.725) for admission on day1, day2 and day3, respectively (Fig. 3B). Comparison of AUC by ROC curve analyses disclosed that FS for day2 was the maximum value for prediction of SAP with a sensitivity of 75.32% and a specificity of 51.76% (Table 2). The number of accurate judgment of SAP in patients whose FS were greater than or equal to each cut-off value, were 204, 207, and 195, respectively (Fig. 3C). FS also had a strong positive correlation with Ranson scoring system and hospital stays (r = 0.549, P = 0.000; r = 0.309, P = 0.000; Fig. 2C and D), which implied that AP patients with high acute fluid sequestration upon admission are more likely to progress to a severe state and require longer hospitalisation.

### Utility of the neutrophil to lymphocyte ratio and fluid sequestration for predicting SAP

Then, to further demonstrate the association of FS, NLR and SAP, an index (NLR-FS) for the prediction of SAP was calculated. The utility of the NLR-FS (NLR day1 and FS day2) for predicting SAP was compared with that of Ranson scoring system (Fig. 3D). ROC analysis revealed that NLR-FS for predicting SAP reached an AUC of 0.810, with high sensitivity of 82.3% and specificity of 60.0%, The AUC of Ranson score predicted SAP was 0.824 with sensitivity of 79.8% and specificity of 76.5% whose calculated cut-off point for prediction was 3. The cut-off levels and evaluative indexes including sensitivity, specificity, PPV and NPV for NLR, FS and the Ranson scoring system are shown in Table 2. The AUC of NLR-FS values were similar to the that of Ranson score (P = 0.6054), indicating NLR-FS also had well capable of distinguishing the SAP occurrence.

## Discussion

Early recognition of disease severity and appropriate therapeutic interventions are extremely crucial for reducing the rates of morbidity and mortality in AP. Patients with a 24-hour delay in admission presented with a 4-fold increase in the risk of death; Prediction of AP severity, especially during the early stage, is still difficult and constitutes a challenge for clinicians^18, 19^. Therefore, the current study was designed to delineate potential predictors to forecast early occurrence of SAP during the clinical course. Our data revealed that NLR and FS were elevated in patients presenting with AP and served as effective markers to monitor the progression of AP. Furthermore, usefulness of NLR and FS for predicting SAP was similar to Ranson score.

NLR is a parameter for the immune status of the host. Neutrophils and lymphocytes are important components of the WBCs and high levels of neutrophil count are associated with a more pronounced inflammatory state. Several investigators also have demonstrated a significant decrease of lymphocyte count in SAP is significantly and independently associated with the development of AP^20, 21^. Additionally, an elevated neutrophil count with a concomitant decreased lymphocyte count has been associated with bacteremia and sepsis but no differece of WBC count^12, 22^. Therefore, NLR may be superior to total WBC or single lymphocyte count in predicting adverse outcomes of AP and may be served as a hallark to discriminate MAP from SAP. To date, there are very few studies have focused on assessing the value of NLR in predicting AP course^15, 17^. The results of these studies confirm the potential for using NLR to predict AP. Basem Azab *et al*. recommend a cut-off value for NLR of ≥4.7 to identify adverse outcome in AP^17^. When we utilized this proposed cut-off in our study, we found that an NLR ≥ 4.7 had accuracy of 55.5–62.8% (Fig. 1C), the highest sensitivity (84–98%) but specificity was only 16–33% (Table 2). Aravind Suppiah *et al*. suggested the optimal cut-offs from ROC curves were 10.6 (day 0), 8.1 (day 1), 4.8 (day 2), respectively but got a very poor positive predictive values (21.2–31.1%)^15^. We considered a trade-off between sensitivity and specificity for clinical use in keeping with other scoring systems such as Ranson score which reported sensitivity of 79.75%, specificity of 76.47% in our cohort.

On the other hand, early antibiotic treatment is correlated with a significant improvement in the prognosis of AP^23^. It is a great divergence of these two studies because carbapenems antibiotics (meropenem and imipenem) can affect leukocyte counts by reducing the inflammatory process^16^. In our study, the broad-spectrum antibiotics, such as third-generation cephalosporins, quinolones, and carbapenems ordinarily used to all patients prophylactically after admission. We may get a different result if we brought the patients who used carbapenems antibiotics into the included criteria; An interesting observation in the current series was the fact that using this measurement (included these 11 patients), the NLR was also significantly elevated in SAP patients with the AUC values were 0.731, 0.754, and 0.673, respectively.

Fluid therapy is considered as a cornerstone of the early management of AP, with several studies and clinical guidelines recommending early and aggressive fluid resuscitation^24, 25^. However, early aggressive fluid therapy may be not associated with improved outcomes or even detrimental^26, 27^. Patients who required fluid therapy partily depended on the status of FS and higher FS in patients may result in hostile fluid therapy. There are very few studies of FS in respect of AP, and mainly focused on the factors associated with level of FS and effects on outcome^8, 11^. These studies indicated that a younger age, alcoholic etiology, presence of systemic inflammatory response syndrome (SIRS), higher hematocrit levels and hyperglycemia were risk factors of developing FS at 48 hours after hospital admission. These patients also have higher susceptibility to local and systemic complications and longer hospital stays^8, 11^. The cut-off value of the FS to predict the severity of AP has not been previously evaluated. In our study, we found that there were statistically significant differences between the MAP and SAP groups in terms of FS for day2 and day3 (P < 0.001). ROC curve analysis revealed there was a significant prediction power of FS for SAP which depended on calculated cut-off value with sensitivity of 62–74% and specificity of 51–57%. Our study also demonstrated that an increased FS on admission corresponds with the development of AP poor outcome, as seen by a positive correlation with Ranson scoring system and hospital stays. Addditionaly, NLR and FS have not been simultaneously assessed as a indicator for SAP prediction. Here, we explored the value of the combination of these two markers for evaluating AP severity and used of ROC curve analysis showed that the AUC of NLR-FS was 0.810, the utility value was superior to NLR or FS alone (p < 0.05) and similar to that of Ranson scoring system (0.810 vs 0.824; P = 0.61).

Current predicting severity of AP is limited to clinical, radiological risk factors, several serum markers and scoring systems. An ideal predictor should show fast, repeatable, inexpensive, minimally invasive and highly accurate results^28^. Our findings suggest that although there is no advantage in time (needs 48 hours), the Ranson criteria, the Acute Physiology and Chronic Health Evaluation (APACHEII) system and the Bedside Index for Severity in Acute Pancreatitis (BISAP) score were more parameters and the complex for quick calculation. NLR-FS could be a low-priced and widely available predictor of SAP. Certainly, the present study has its inherent limitations that should be considered. First, it is a retrospective collection of fluid administration and losses, there could failure in the recording of unconspicuous intake and output values. A standard calculation method of fluid output were including the total volumes of vomitus, urine, and stool. An additional 500 ml was added to the output if patient had a temperature ≥37.8 °C per day^8^. we also did not calculate the insensible fluid losses based on body weight^29^; Second, information on SAP outcome, such as mortality and organ failure were not available in our analyses; Third, the study came from one center and the samples of patients are relatively small suggesting restricted application of the results; In order to objectively evaluate the significance of these parameters, a multicenter prospective study with a larger patient cohort is needed.

## Materials and Methods

### Study population

**Figure 4:**
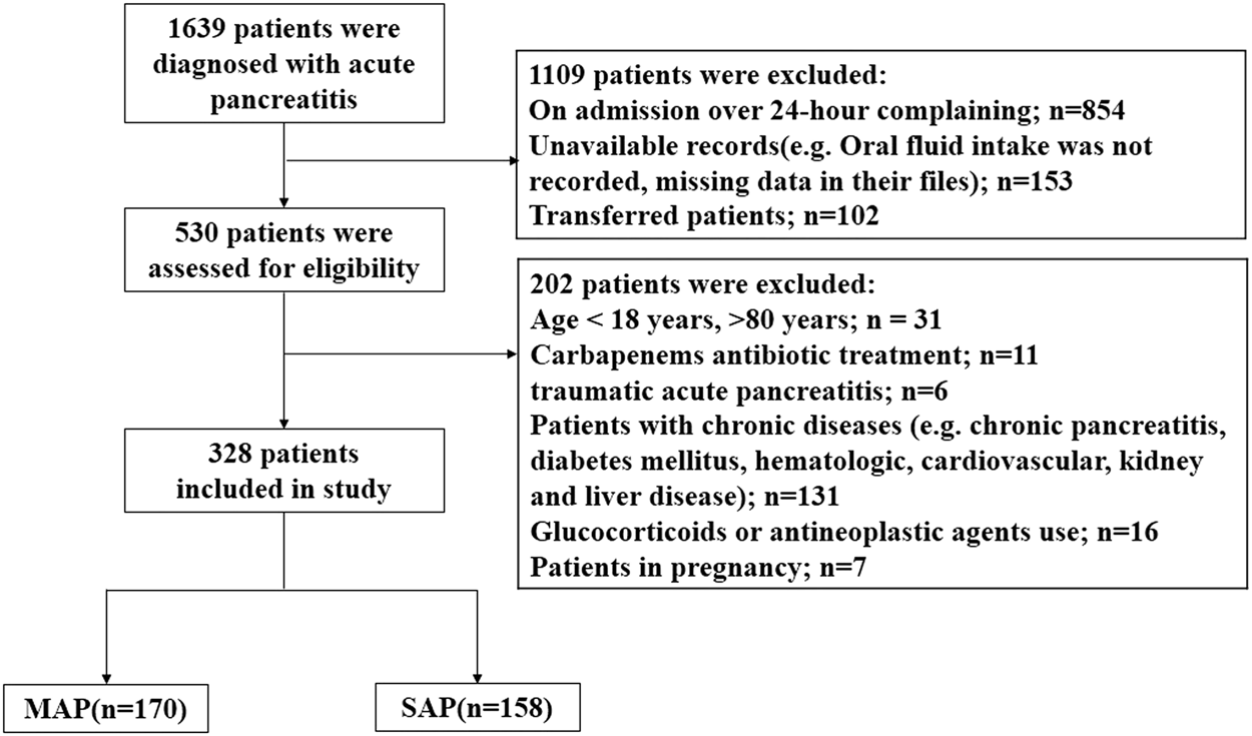
Screening and grouping of the study cohort.

We extracted patient data from a review of inpatient medical records. A total of 1639 patients with AP presenting to the Department of Gastroenterology, Union Hospital, Wuhan, China over the 4-year study period (between January 2012 and January 2016) were included. Severity degree of patients was defined according to the new revised classification of acute pancreatitis 2012^30^. However, to enable comparison with previous studies, patient outcomes were classified into mild acute pancreatitis (MAP) and SAP (including MSAP). The inclusion criteria required the clinical diagnosis of AP and the interval between occurrence of symptoms and on admission was within 24 h. We excluded patients with chronic diseases, such as heart failure and end stage renal and hepatic disease, as these might confound evaluation of FS. We also excluded patients with carbapenems antibiotic treatment such as meropenem and imipenem, as these might affect leukocyte counts^16^. Other criteria for exclusion, such as patients who were transferred from other institutions, those with glucocorticoids use or those with incomplete records were described detailedly in Fig. 4; and fininally 328 patients were eligible for further analysis. The study was approved by the Ethics Committee of Tongji Medical College, Huazhong University of Science and Technology (No: IORG0003571). The study methods were carried out in accordance with approved guidelines and all subjects provided written informed consent prior to study enrolment. Their data had been anonymized and de-identified.

### Data collection

To determine the value of admission NLR and FS, the following data were retrospectively collected for each patient: demographics (i.e., gender, age), process measures (i.e., admission antibiotics, hospital stays), complete blood count (i.e., White blood cell, neutrophil and lymphocyte), serum biochemical indexes (i.e., amylase, lipase, blood glucose, C-reactive protein (CRP), aspartate aminotransferase (AST), alanine aminotransferase (ALT), creatinine and calcium level), lifestyle factors, including tobacco smoking, alcohol consumption. The NLR was calculated the ratio between the neutrophil and lymphocyte counts on illness onset within 24 h (day0), 48 h (day1) and 72 h (day2). The volume fluid administered and urine volume were recorded from initial presentation in the hospitalization using nursing administration documentation. FS was calculated by subtracting the total fluid output from the total fluid input. On admission within 24 h, 48 h and 72 h were labelled as day1, day2 and day3 for FS, respectively. The volumes of FS for day2 included the FS for day1. Similarly, the volumes of FS for day3 included the FS for day1 and day2. At the time of data abstraction, collectors were blinded to the outcomes being investigated.

### Statistical analysis

Continuous variables were presented as mean ± standard deviation (SD). Frequencies and proportions (%) were used to describe categorical data. The Mann–Whitney U-test or the Pearson’s chi-square test was used to evaluate differences between two groups. The area under the curve (AUC) of receiver operator characteristic (ROC) curve was used to evaluate the discriminative ability of NLR and FS in AP. This was also used to assess the optimal cut-off, by showing the trade-off between sensitivity and specificity. Subsequently, for determination of combined variable in predicting SAP, a logistic regression analysis was performed. Correlation analysis was assessed by Spearman’s rank test. Statistical analyses were performed with SPSS software 19.0 and MedCalc software 15.6. A *P*-value < 0.05 was defined as statistically significant.
